# Temperature Sensitivity of Soil Organic Carbon Mineralization along an Elevation Gradient in the Wuyi Mountains, China

**DOI:** 10.1371/journal.pone.0053914

**Published:** 2013-01-14

**Authors:** Guobing Wang, Yan Zhou, Xia Xu, Honghua Ruan, Jiashe Wang

**Affiliations:** 1 Faculty of Forest Resources and Environmental Science, and Key Laboratory of Forestry and Ecological Engineering of Jiangsu Province, Nanjing Forestry University, Nanjing, Jiangsu, China; 2 Nanning Landscape Administration Bureau, Nanning, Guangxi, China; 3 Department of Microbiology and Plant Biology, University of Oklahoma, Norman, Oklahoma, United States of America; 4 Administrative Bureau of Wuyishan National Nature Reserve, Wuyishan, Fujian, China; DOE Pacific Northwest National Laboratory, United States of America

## Abstract

Soil organic carbon (SOC) actively participates in the global carbon (C) cycle. Despite much research, however, our understanding of the temperature sensitivity of soil organic carbon (SOC) mineralization is still very limited. To investigate the responses of SOC mineralization to temperature, we sampled surface soils (0–10 cm) from evergreen broad-leaf forest (EBF), coniferous forest (CF), sub-alpine dwarf forest (SDF), and alpine meadow (AM) along an elevational gradient in the Wuyi Mountains, China. The soil samples were incubated at 5, 15, 25, and 35°C with constant soil moisture for 360 days. The temperature sensitivity of SOC mineralization (Q_10_) was calculated by comparing the time needed to mineralize the same amount of C at any two adjacent incubation temperatures. Results showed that the rates of SOC mineralization and the cumulative SOC mineralized during the entire incubation significantly increased with increasing incubation temperatures across the four sites. With the increasing extent of SOC being mineralized (increasing incubation time), the Q_10_ values increased. Moreover, we found that both the elevational gradient and incubation temperature intervals significantly impacted Q_10_ values. Q_10_ values of the labile and recalcitrant organic C linearly increased with elevation. For the 5–15, 15–25, and 25–35°C intervals, surprisingly, the overall Q_10_ values for the labile C did not decrease as the recalcitrant C did. Generally, our results suggest that subtropical forest soils may release more carbon than expected in a warmer climate.

## Introduction

The dynamics of soil organic carbon (SOC) mineralization is an important issue in global climate change [1]–[2], as SOC mineralization plays an important role in regulating global atmospheric CO_2_ concentration. Many factors, such as soil temperature [3]–[4], soil structure [5], soil moisture [6]–[7], characteristics of soil micro-organisms and microbial community [8], and substrate quality and availability [9], influence SOC mineralization. In the context of global warming, however, it is particularly important to understand the temperature sensitivity of soil carbon (C) mineralization. It is anticipated that ecosystems may exert a positive feedback to the rising temperatures because of the stronger response of decomposition to temperature than that of net primary productivity [10]–[11]. If the amount of plant-derived C incorporated into soil exceeds the C loss through decomposition, on the other hand, a negative feedback may occur.

In order to investigate the SOC mineralization-temperature relationship, temperature response functions are essential to simulate the effects of global warming on the mineralization of soil C pools [12]–[13] and the potential feedback to current global warming [1], [14]. However, a majority of the simulation models used to predict the fate of the soil C stock under global warming utilize the same coefficient for simplicity as the indicator of the temperature sensitivity of SOC decomposition regardless of the ecosystem types, bio-climatic zones, or the stability of the organic matter pools [3], [15]. Previous studies indicate that the temperature sensitivity of SOC mineralization varies, depending on the types of SOC and the extent of SOC being mineralized. Additionally, knowledge on whether the labile C has relatively lower temperature sensitivity than that of the recalcitrant C is still limited [16]–[21], constraining our accuracy in predicting feedbacks of potential C dynamics to future climate change.

Global mean temperature is predicted to increase another 2–7°C by the end of this century [22] and is anticipated to significantly influence microbial mineralization of soil organic matter [23]. Laboratory incubations of soil provide us a useful way to study the intrinsic temperature sensitivity of SOC decomposition with few confounding impacts of the many factors influencing Q_10_ values in field conditions [24]. Some earlier studies proved that the temperature sensitivity of decomposition decreased with increasing temperature given lower Q_10_ values at higher temperatures [3], [25]. Others showed that Q_10_ values varied largely across the range of temperatures, which were low at low temperature interval (0–10°C), increased at median temperature interval (10–20°C), and then decreased at high temperature interval (20–30°C) [26]. Studying the temperature sensitivity of SOC mineralization at different temperature intervals around 15°C (because the mean annual temperature is 15°C for the Wuyi Mountains) is particularly importantly important for understanding the dynamics of soil C pools under warmer temperatures. At present, much attention has been paid to the responses of SOC mineralization to temperature changes in the tropical, temperate, and boreal regions [27]–[29]. Studies in the subtropical regions are rare but of great importance, especially along an elevational gradient because temperature changes in mountains along an elevation can be similar to that caused by latitudinal gradients [26], [30].

The variations along an elevation in mountainous areas provide a unique opportunity to study the SOC mineralization-temperature relationship [31]–[33]. In particular, soils along elevation gradients are sensitive to multiple environmental factors that have interacted over long periods of time and they are suitable for testing the effect of warmer temperatures on SOC mineralization [33]–[36]. Compared to the regions at the same latitude in the world, the Wuyi Mountains have the largest and the most well-preserved subtropical forest ecosystems. Moreover, elevational gradients of temperature changes could resemble those observed along latitudinal gradients [26]. We studied SOC mineralization-temperature relationship in this study and the specific aims were to: (1) examine the variation in the Q_10_ values of SOC mineralization along with the increasing extent of SOC being mineralized; and (2) investigate the effects of elevation and incubation temperature intervals (5–15°C, 15–25°C, and 25–35°C) on Q_10_ values.

## Materials and Methods

### Site Description

Our experimental sites are located in the Wuyishan National Nature Reserve Area in Fujian Province (27°33′–27°54′N, 117°27′–117°51′E), a 56,527 ha forested area in the southeastern China. Mean annual temperature (MAT), relative humidity, and annual precipitation (AP) for this area are 18°C, 83.5%, and 2,000 mm, respectively. Four typical vegetation types are distributed along the elevation gradient: evergreen broad-leaf forest (EBF), coniferous forest (CF), sub-alpine dwarf forest (SDF), and alpine meadow (AM) [37]–[38].

The first site is located in a 1,175 ha subtropical EBF at 500 m above sea level (asl) with the AP of 1,700 mm. MAT was 18°C [37]–[38]. *Castanopsis carlesii* with an average height of 14.7 m was the prevailing tree species at this site. The second site is a temperate CF, locating at an elevation of 1,150 m (asl), with the MAT of 14.5°C [39] and the AP of 2,000 mm [38]. The forest was dominated by *Pinus tanwanensis* trees with a mean diameter of 22 cm at breast height (DBH). The third site at an elevation of 1,750 m (asl) is a SDF with an AP of 2,200 mm and the MAT of 11.2°C [38]–[39]. The dominant tree species at this site were *Symplocos paniculata* and *Stewartia sinensis* with the average tree height 4.5 m. The fourth site, AM, is located at an elevation of 2,150 m (asl) and was close to the highest mountain in the southeastern China. The AP was 3,100 m and the MAT was 9.7°C [38]–[39]. The site was covered by grasses with an average height of 25 cm and the dominant species were *Calamagrostis brachytricha*, *Miscanthus sinensis*, and *Lycopodium clavatum*. Detailed site conditions are shown in Table 1.

**Table 1:** Table **1.** Site conditions.

Site	Elevation(m)	AP (mm)	MAT (°C)	Soil Moisture(%)	Soil Temperature (%)	SOC (g kg^−1^)	TN (g kg^−1^)	C/N	pH	Bulk density (g cm^−3^)
EBF	500	1,700	18	22.43±0.12^d^	16.77±0.11^a^	44.78±0.44^d^	5.46±0.04^c^	8.20±0.03^c^	4.67±0.05^b^	0.962±0.10^a^
CF	1,150	2,000	14.5	36.52±1.83^c^	12.70±0.05^b^	59.63±2.92^c^	5.27±0.02^d^	11.31±0.54^b^	4.10±0.02^d^	0.795±0.06^b^
SDF	1,750	2,200	11.2	51.91±1.34^b^	11.83±0.09^c^	96.27±1.75^b^	8.05±0.02^b^	11.96±0.21^b^	4.55±0.02^c^	0.708±0.04^c^
AM	2,150	3,100	9.7	55.47±0.53^a^	11.19±0.06^c^	140.45±3.66^a^	10.06±0.02^a^	13.96±0.34^a^	4.83±0.01^a^	0.667±0.05^c^

Note: AP, annual precipitation; MAT, mean annual temperature; SOC, soil organic carbon; TN, total nitrogen; EBF, evergreen broad-leaf forest; CF, coniferous forest; SDF, sub-alpine dwarf forest; AM, alpine meadow. Values are mean±SE. Different letters within a column indicate significant differences at *P*<0.05. Datasets of AP are obtained from a previous study^37^.

### Experimental Design and Soil Sampling

Four replicate plots (25 m×30 m) were randomly set up in each forest (EBF, CF, and SDF) and in AM along the elevational gradient at the Wuyi Mountains. In late April, 2007, we randomly collected surface soil samples (0–10 cm) from all the 25 m×30 m plots using a 2 cm-diameter soil corer. Each soil sample was a composite of twenty cores. Samples were immediately sieved (<2 mm) to remove soil fauna, rocks and fine roots, thoroughly hand-mixed, placed in plastic bags and transported in several coolers to our laboratory at the Nanjing Forestry University. We kept the soil samples in a refrigerator at 5.0°C before being used for incubation. A small part of each soil sample was air-dried, ground, and sieved through a 0.25 mm sieve to measure SOC and other chemical properties.

### Soil Incubation and Chemical Analyses

The soil samples (100 g) went through a two-week pre-incubation at 15°C and 75% of field capacity to avoid the “pulsing effect”, which may result in a rapid mineralization of SOC. Then, they were incubated in 1 L Mason jars at 5, 15, 25, and 35°C (±1°C) for 360 days in four LRH-450 incubators (Medicine Machinery Co. Ltd., Shanghai, China). Meanwhile, controls (without soil samples) were also incubated in the incubators. Small vials (50 ml, with no lids) containing 30 ml of 1 M NaOH solution were periodically placed in each Mason jar to trap respired CO_2_ [40]. Samples were taken after 7, 14, 21, 35, 49, 63, 78, 93, 110, 130, 150, 170, 190, 210, 235, 260, 285, 310, 335 and 360 days by removing the NaOH vials. To calculate the C mineralization rate, the amount of CO_2_ was determined by titration of the NaOH with 1 M HCl to pH 8.3 in the presence of BaCl_2_. Then, the mason jars were flushed with compressed air to allow replenishment of O_2_ after each interval and deionized water was added to maintain moisture at 75% of field capacity.

Soil organic carbon (SOC), total nitrogen (TN), and total sulfur (TS) were measured using a CNS Macro Elemental Analyzer (Elementar Analysen Systeme GmbH, Germany). Soil moisture was determined by oven-dry soil samples at 105°C and was expressed on a dry mass basis. Soil pH was measured in soil/H_2_O suspension (1∶2.5, w/w) with a 716 DMS Titrino pH meter (Metrohm Ltd. CH.-901 Herisau, Switzerland) fitted with a glass electrode. Soil bulk density was determined by soil coring.

### Statistical Analysis

The temperature sensitivity (Q_10_) of SOC mineralization during the incubation was calculated according to Xu et al. [37]:
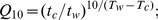
where *t_c_* and *t_w_* are the time required to respire a given amount of soil C at relatively cold (*T*_c_) and warm (*T*_w_) temperatures during incubation. The first 8% of the initial C was considered to be relatively labile and the rest to be recalcitrant [18], [37]. The Q_10_ values for the labile C pool were estimated by dividing the time taken to mineralize the first 1% of initial C at cold temperature (e.g. 15°C) by that at warm temperature (e.g. 25°C). For the recalcitrant organic C (ROC) pool, Q_10_ values were determined using the time taken to respire an additional 1% of initial C after 8% of initial C was decomposed. Q_10_ values based on the calculation of the time need to mineralize the same amount of C at different incubation temperatures could ensure that we were comparing the SOC being mineralized at the same extent and eliminating the confounding effect arisen from the changes in substrate availability with time.

We used one-way ANOVA to identify the differences in soil chemical properties, cumulative SOC mineralized during the whole incubation, and the Q_10_ values. Two-way ANOVA analyses were performed to examine the effects of elevation and temperature intervals of incubation for the Q_10_ values, including Q_10-labile_ and Q_10-recalcitrant_ values. All statistical analyses were conducted using SPSS 16.0 software (SPSS Institute Inc., Chicago, IL, USA).

## Results

### SOC Mineralization

**Figure 1:**
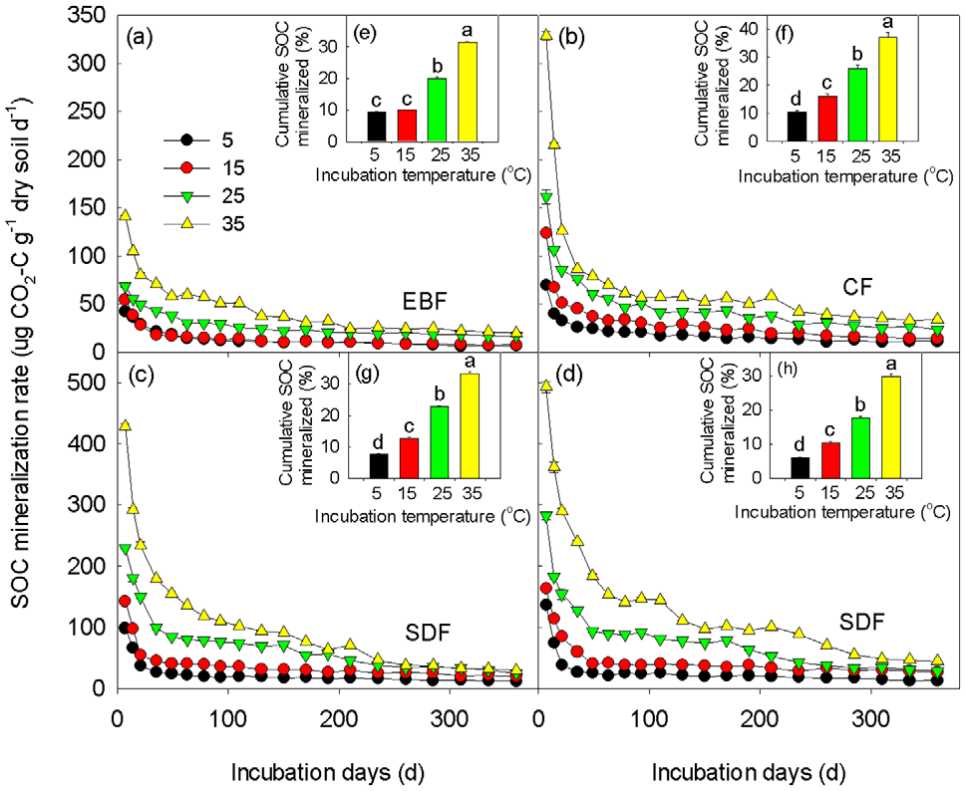
Variation in the rates of SOC mineralization during the whole incubation at different incubation temperatures (5, 15, 25, and 35°C) in EBF (a), CF (b), SDF (c), and AM (d). Inserted panels e for EBF, f for CF, g for SDF, and h for AM show the cumulative percent of SOC mineralized during the whole incubation. Different letters indicate significant differences in the cumulative percent of SOC mineralized among different incubation temperatures at *P*<0.05. Values are Mean±SE. EBF, evergreen broadleaf forest; CF, coniferous forest; SDF, sub-alpine dwarf forest; AM, alpine meadow.

Laboratory incubation temperatures significantly influenced the mineralization rates of SOC (Fig. 1). Both the mineralization rates (Fig. 1A, 1B, 1C, 1D) and the proportion of the cumulative C mineralized during the whole incubation period (Fig. 1E, 1F, 1G, 1H) increased with increasing incubation temperatures. In EBF during the first incubation cycle (the first 7 days), for example, the average mineralization rate at 35°C was 3.34, 2.60, and 2.06 times higher than those at 5, 15, and 25°C. The mineralization rates of SOC declined substantially over the entire incubation period across the incubation temperatures and the four elevational vegetation types. The incubation temperatures significantly affected the cumulative C mineralized that increased with increasing temperatures across the four vegetation types. The mineralization rates of SOC decreased and leveled off as the incubation proceeded (Fig. 1). After 360 days of incubation, at least 6.15% of the initial C, found in the soil samples incubated at 5°C in AM, had been mineralized.

### Sensitivity of SOC Mineralization to Temperatures

**Figure 2:**
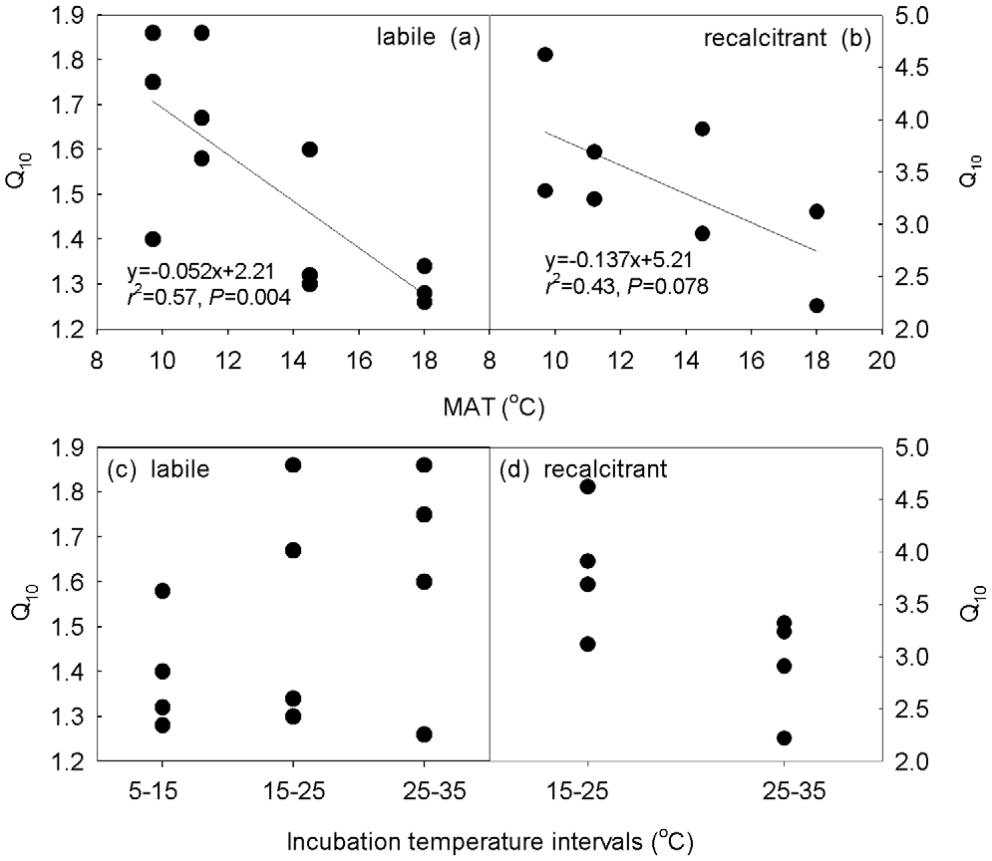
Relationship of Q_10_ values with mean annual temperature (MAT) of the different elevations (a, b) and incubation temperature intervals (c, d).

**Figure 3:**
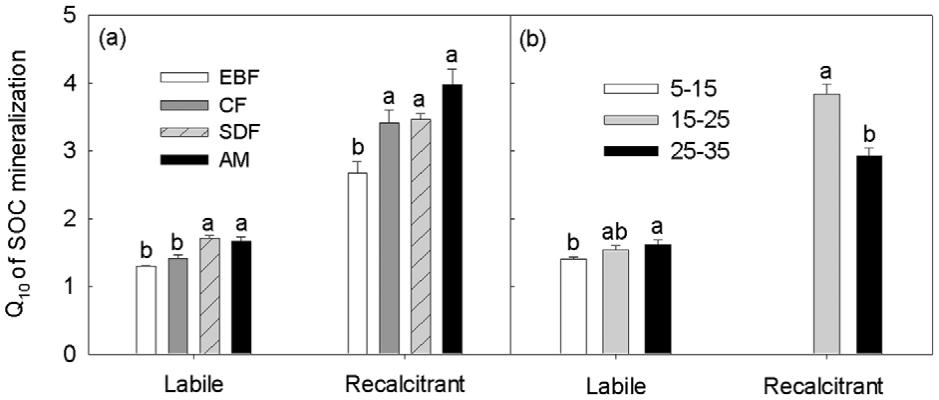
The temperature sensitivity of SOC mineralization for both the labile and the recalcitrant SOC mineralization along the elevation in the four vegetations (a) and at different incubation temperature intervals (b). Values are Mean±SE. Different letters stand for significant difference at *P*<0.05. Asterisks indicate the statistical difference at *P*<0.01. Missing value in Figure 2C at 5–15°C is because some soils incubated at 5°C did not respire 9% of the total C during the incubation period. LOC: the SOC can be easily decomposed by microorganisms, resulting from the fresh residues such as plant roots and living microbes; ROC: the SOC that is resistant to decay, such as cellulose, humus, and tannin.

The temperature sensitivity of SOC mineralization (Q_10_) increased with increasing incubation time (Table 2). This phenomenon held true across all the incubation temperature intervals along the elevation gradient. Both the elevation and the incubation temperature intervals had significant effects on Q_10_ values at different time points during the incubation (all *P*<0.05, Table 2). Q_10_ values increased greatly with increasing elevations both for the labile and recalcitrant SOC (*P* = 0.004 and 0.078, respectively, Fig. 2A, 2B). With increasing incubation temperature intervals, however, Q_10_ values did not linearly increase (all *P*>0.05, Fig. 2C, 2D). In EBF and AM, specifically, Q_10_ values did not changed from the 15–25°C interval to the 25–35°C interval (Table 2, *P*>0.05). Overall, the Q_10_ for the recalcitrant SOC mineralization was much higher than that of the labile (*P*<0.05, Fig. 3A). Surprisingly, we found that the Q_10_ values for the labile SOC mineralization were higher at higher temperature intervals (Fig. 3B). For the recalcitrant SOC mineralization, however, the Q_10_ values decreased with increasing temperature intervals.

**Table 2:** Results of two-way ANOVA for Q_10_ values at different temperature intervals in the four elevational vegetation communities.

Vegetation	Temp. interval (°C)	1%-Labile	1–2%	5–6%	8–9%-Recalcitrant
EBF	5–15	1.28±0.03	1.22±0.05	2.01±0.10	–
	15–25	1.34±0.03	1.70±0.03	2.92±0.07	3.12±0.12
	25–35	1.26±0.03	2.03±0.01	2.22±0.07	2.22±0.07
CF	5–15	1.32±0.08	1.42±0.21	1.97±0.03	–
	15–25	1.30±0.02	1.72±0.05	2.15±0.03	3.91±0.08
	25–35	1.60±0.12	2.50±0.20	3.00±0.09	2.91±0.11
SDF	5–15	1.58±0.07	1.66±0.06	1.93±0.05	–
	15–25	1.67±0.01	2.55±0.15	2.60±0.09	3.69±0.09
	25–35	1.86±0.04	2.03±0.05	2.70±0.09	3.24±0.04
AM	5–15	1.40±0.03	1.88±0.03	2.34±0.06	–
	15–25	1.86±0.07	2.45±0.05	2.43±0.02	4.62±0.12
	25–35	1.75±0.04	2.31±0.07	2.94±0.17	3.32±0.06
Source of variation					
Elevation		*	*	*	*
Temp. interval		*	*	*	*
Elevation×Temp. interval		*	*	*	*

Note: EBF, evergreen broadleaf forest; CF, coniferous forest; SDF, sub-alpine dwarf forest; AM, alpine meadow. An asterisk represents significant effect of elevation or incubation temperature interval on Q_10_ values at *P*<0.05. 1%, 1–2%, 5–6%, and 8–9% stand for the mineralization of the first, second, sixth, and ninth percent of SOC. Missing values for the 8–9% at 5–15°C are because some soils incubated at 5°C did not respire 9% of the total C during the incubation period.

## Discussion

### Comparisons of SOC Mineralization

Climatic conditions are known to affect the accumulation of soil carbon with the highest soil carbon stocks being generated in cold and humid biomes [41]. The altitude, similar to latitude, produces strong gradients in soil carbon stocks [42]. In this study, soil carbon stocks ranked as AM>SDF>CF> EBF (Table 1). The marked spatial differences in temperature and water along elevational gradients in the Wuyi Mountains are probably responsible for the strong observed response of the soil carbon stocks. In general, SOC mineralization followed the similar pattern for all soil samples showed in Figure 1, which was fast during the first 55 days and then slowed down and kept relatively stable in the next 305 days. With increasing incubation time, a decline in SOC mineralization rates was widely observed [19]–[20]. This indicated that the labile C was progressively depleted and the proportion of recalcitrant C became larger. SOC mineralization and the amount of SOC mineralized during incubation increased with increasing incubation temperatures. This is in line with previous studies [19], [37] and a general expectation that warmer temperatures would accelerate the SOC mineralization [43].

With the rising concentration of greenhouse gases in the atmosphere, increases in global temperature are expected to continue and become even more pronounced.

### Comparisons of *Q*_10_ at Different Mineralization Levels

We found that the temperature sensitivity of SOC mineralization largely increased with the increased extent of SOC mineralization (Table 2). This is reasonable that as mineralization progressed over time, the contribution of recalcitrant C gradually increased, which has relatively higher sensitivity to temperature changes. Study by Zhu and Cheng [44] also found that Q_10_ values of SOC decomposition increased with increasing decomposition of SOC, which were estimated using the same method [18], [37]. The differences in the response of SOC mineralization to temperatures found in our study indicated a shift to the decay of biochemically recalcitrant C from labile C. In contrast to our results, a previous study suggests that SOC being mineralized to different extent responds to temperature changes in a similar way based on the averaged Q_10_ values of the decomposition of intact and root-free soil samples from different layers (0–10, 20–30 cm) [20]. However, the dynamics of SOC decomposition are likely to be quite different between intact and root-free soils originated from different layers with substantially different factors such as substrate availability [1] and physical protection [45], preventing us from directly comparing the results. The limitation is that we did not analyze the microbial community structure during the incubation, which may play a role in influencing the Q_10_ values. However, changes from labile SOC to recalcitrant SOC were believed to be the dominant factor in regulating the increase in Q_10_ values [2].

### Impact of Elevation and Temperature Intervals on Q_10_

The temperature sensitivity of SOC mineralization significantly increased along the elevation gradient, both for the labile and the recalcitrant C mineralization (Table 2; Fig. 2A, 2B, Fig. 3A). Previous studies have pointed out that changes in elevation can result in alterations in biological and ecological factors, such as forest type and plant community structure, soil microbes, soil temperature and moisture, precipitation, and nutrients [15], [35], [46], consequently affecting the mineralization of SOC. For example, high Q_10_ values at high elevations might be related to the microbial community structure that originated from relatively colder temperatures (Table 1) and had higher metabolic efficiency [47]. Though our previous study at the same site [37] found that Q_10_ values for the labile C did not vary significantly along the elevation, the results of the two studies are not contradictory. First, soil samples were not taken at the same time of each year, one in March, 2006 and the other in late April, 2007 of this study. Second, soil moisture was controlled at different level, 60% vs. 75% (this study). Previous studies showed that soil moisture alone or its interaction with incubation temperatures would influence the rates and Q_10_ values of SOC mineralization, and would further affect the Q_10_ values of SOC mineralization [6], [7], [48]. Most importantly, results based on one-way ANOVA showed that Q_10_ values for the 15–25°C interval of the two studies did not differ significantly from each other (*P* = 0.10, n = 4).

The temperature sensitivity of SOC mineralization was found to be temperature dependent in the Wuyi Mountains. For example, our results showed that the Q_10_ values for the labile C mineralization increased from 5–15°C interval to 15–25°C interval (Fig. 3B). Similar results have been reported [26], [49] that calculated Q_10_ values were higher for the temperature range of 10–20°C than for the 0–10°C°C range. Surprisingly, the Q_10_ values on average did not decrease at the 25–35°C interval (*P*>0.05, Fig. 3B) though many studies done earlier indicated that the temperature sensitivity of decomposition decreased with increasing temperatures [3], [50]. It is well known that temperature and moisture are much more important than other factors in affecting the mineralization process of organic matter [51]. In our study, soil moisture was kept at 75% of field capacity but, this value may not be optimal for SOC mineralization at all incubation temperatures. Moreover, the interactive effect of moisture with incubation temperatures would differ among soils [7], [49]. On the other hand, the Q_10_ values for the recalcitrant C mineralization decreased from 15–25°C interval to 25–35°C interval (Fig. 3B). The different responses of labile and recalcitrant C mineralization to temperatures may be attributable to the changes in the microbial community structures and the physiochemical properties of organic matter themselves being incubated. Additionally, the previous finding by Xu et al. [37] that recalcitrant C was much more sensitive to the changes in temperature at the same study site was further confirmed by this study (Table 2, Fig. 3A). Although several studies found that the temperature sensitivity of the labile C could be higher [16], [17] or similar [20], [21] to that of the recalcitrant C, more and more studies have demonstrated that recalcitrant C is more temperature sensitive [2], [18], [19], [23], [47], in accordance with kinetic theory based on chemical reactions.

These suggest that warmer temperatures may accelerate CO_2_ effluxes from soil via organic carbon mineralization in the subtropical region because (1) both the labile and recalcitrant C mineralization were sensitive to temperatures; (2) the temperature sensitivity of recalcitrant C mineralization was higher than that of labile C; and (3) most importantly, the temperature sensitivity of labile C mineralization increased with increasing temperature given a higher Q_10_ value at higher temperature range, 15–25°C, which projected regional temperature would fall into this range [22]. In considering global warming, the role of subtropical forests on the release of soil carbon under rising atmospheric temperature can thus not be ignored.
